# Targeted Transferable Machine-Learned Potential for Linear Alkanes Trained on C_14_H_30_ and Tested for C_4_H_10_ to C_30_H_62_

**DOI:** 10.1021/acs.jctc.4c01793

**Published:** 2025-03-27

**Authors:** Chen Qu, Paul L. Houston, Thomas Allison, Joel M. Bowman

**Affiliations:** † Independent Researcher, Toronto, Ontario M9B0E3, Canada; ‡ Department of Chemistry and Chemical Biology, Cornell University, Ithaca, New York 14853, United States; § Department of Chemistry and Biochemistry, Georgia Institute of Technology, Atlanta, Georgia 30332, United States; ∥ National Institute of Standards and Technology, 100 Bureau Drive, Gaithersburg, Maryland 20899, United States; ⊥ Department of Chemistry and Cherry L. Emerson Center for Scientific Computation, Emory University, Atlanta, Georgia 30322, United States

## Abstract

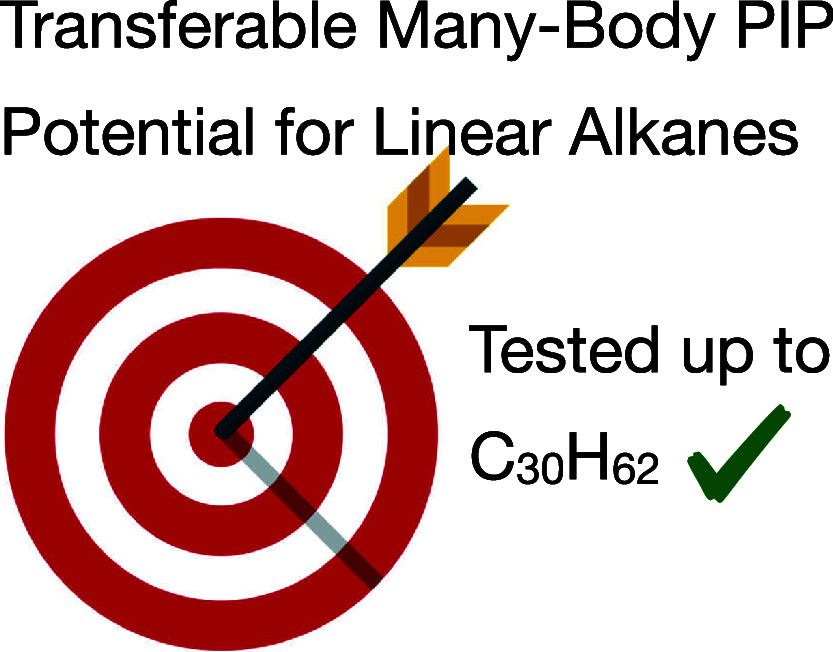

Given the great importance of linear alkanes in fundamental and applied research, an accurate machine-learned potential (MLP) would be a major advance in computational modeling of these hydrocarbons. Recently, we reported a novel, many-body permutationally invariant model that was trained specifically for the 44-atom hydrocarbon C_14_H_30_ on roughly 250,000 B3LYP energies (Qu, C.; Houston, P. L.; Allison, T.; Schneider, B. I.; Bowman, J. M. *J. Chem. Theory Comput.***2024**, *20*, 9339–9353). Here, we demonstrate the accuracy of the transferability of this potential for linear alkanes ranging from butane C_4_H_10_ up to C_30_H_62_. Unlike other approaches for transferability that aim for universal applicability, the present approach is targeted for linear alkanes. The mean absolute error (MAE) for energy ranges from 0.26 kcal/mol for butane and rises to 0.73 kcal/mol for C_30_H_62_ over the energy range up to 80 kcal/mol for butane and 600 kcal/mol for C_30_H_62_. These values are unprecedented for transferable potentials and indicate the high performance of a targeted transferable potential. The conformational barriers are shown to be in excellent agreement with high-level ab initio calculations for pentane, the largest alkane for which such calculations have been reported. Vibrational power spectra of C_30_H_62_ from molecular dynamics calculations are presented and briefly discussed. Finally, the evaluation time for the potential is shown to vary linearly with the number of atoms.

## Introduction

Machine-learned potentials (MLPs) offer the promise of providing “first-principle” approaches for the full-span of molecular dynamics simulations.^1^ Among the panoply of machine learning (ML) methods,^2^ permutationally invariant polynomials (PIPs)^3−5^ have been used for nearly 20 years to develop precise, high-dimensional potential energy surfaces (PESs) for molecules,^6−9^ clusters,^10−12^ many-body terms for water potentials,^13−16^ and atom-centered many-body representations for materials^17^ and molecular force fields.^18^ The precision of a PIPs PES for “rMD17 ethanol” was shown to be as good as the best performing ML methods and to be substantially faster (factors of 10 or more)^19^ than all the ML methods considered, i.e., GAP-SOAP,^20^ ANI,^21^ DeePMD,^22^ sGDML,^23,24^ PhysNet,^25^ KREG,^26^ and pKREG.^27^ Similar factors in speed differences and high precision for PIPs were recently reported for 21-atom aspirin.^28^ In addition, PIPs have been used to fit more extensive and complex data sets than are given in the rMD17 data set^29^ for molecules with 10 or more atoms. Examples include formic acid dimer,^30^ glycine,^31^*N*-methylacetamide,^32^ acetyl acetone,^33^ and tropolone,^34^ 21-atom aspirin^28^ and most recently 44-atom C_14_H_30_.^35,36^ PIPs have also been used effectively and extensively as inputs to Neural Network^37−39^ and Gaussian Process Regression^40^ machine learning for potentials. To date roughly 300 such PESs have been reported.

In addition to these molecule-specific MLPs, there is the aspiration to develop transferable MLPs for a large chemical compound space. Such potentials could provide an alternative to widely used universal classical force fields, e.g., CHARMM,^41^ AMBER,^42^ and MM3.^43^ A number of approaches can be used toward this goal. In principle, atom-centered representations of a potential appear inherently transferable. This follows directly from the starting position of this approach that the total (Born–Oppenheimer) energy is given by the sum of atomic energies. Of course, there is no unique atomic energy, and this is not assumed in this atom-centered approach. So, in each application the atomic energies must be “trained” using a parametrization of the energy, typically the atomic electronic density for a given molecular data set. Then the energy functional of the density is fit using a data set of ab initio molecular energies and perhaps forces. Depending on the nature of the data set and also the nature of the molecular interaction, i.e., covalent or noncovalent, the training may lead to a transferable representation. This approach is general and applies to covalent and noncovalent interactions.

The seminal atom-centered approach of Behler and Parrinello used nonlinear, neural network regression for training on a data set of DFT energies of Si.^44^ This general approach, in which the energy of each atom is represented by a neural network, has been extended by other groups, notably Roitberg and co-workers, with the aim of creating a universal transferable machine-learned force field for any compound containing C, H, N, and O atoms. This group showed the major improvement in accuracy for the ANI-1 method for a large class of molecules for DFT energies relative to semiempirical methods.^21^ An excellent review of this approach, with a focus on reaction energies, along with discussion of other ML methods has just appeared.^45^ Typical precision metrics, i.e., RMSE and MAE are 3 kcal/mol# for energies and 1.6–2.1 kcal mol^–1^ bohr^–1^ for forces.^46^ Note, unless indicated otherwise, these metrics are not per atom. Also, we note recent and relevant work using the atom-centered “Atomic Cluster Expansion” (ACE) approach.^47^ This general approach has been extended in several important ways by Csanyi and co-workers, starting with aPIPs,^17,18^ then moving to “ACE”^48^ and currently to the nonlinear Neural Network version “MACE”.^49−51^ Also noteworthy are atom-centered approaches from Tkatchenko and co-workers.^29,52^

Nonatom-centered approaches to MLPs do not appear, at first glance, to be transferable. A large class of such approaches exist (a sample of these is listed above), using global descriptors such as Morse variables, *y*_*ij*_, i.e., transformed internuclear distances exp(−*r*_*ij*_/*a*), where *a* is the Morse range parameter. One example of these approaches is PIPs, which use Morse variables as the inputs. These are very successful both in terms of precision and speed in PIP potentials^3^ or using PIPs as inputs to neural networks^37,53^ and Gaussian Process Regression.^40^

However, for noncovalent interactions, a standard many-body approach does enable these nonatom-centered methods to be transferable, albeit in a targeted fashion. Prominent examples of this approach are the CCSD(T) quality water potentials q-AQUA^15^ and MB-Pol(23);^16^ these include 1-b, 2-b, 3-b, and 4-b interactions, where “b” represents the water monomer.

For covalent interactions the territory is new. Paesani and co-workers have taken a significant step in this direction recently for small hydrocarbons, using PIPs.^9^ More recently, we reported two PIPs-based potentials for C_14_H_30_, one of which, denoted MB-PIP PES, is manifestly transferable.^35^ Since this PES was specifically trained on the linear alkane C_14_H_30_, the transferability is targeted to all linear alkanes. This paper presents tests of the precision and robustness of this transferable MB-PIP PES for 26 alkanes ranging from butane to the 92-atom C_30_H_62_. Small changes were made for this PES, and to make the distinction between the original MB-PIP PES for C_14_H_30_ and the new transferable one, we introduce the notation “t-MB-PIP” for the general transferable PES.

The paper is organized as follows. First, a short review of the general many-body PIP approach is given, including a recap of MB-PIP PES trained for C_14_H_30_. Details of the data used for the testing of the t-MB-PIP PES are then given followed by results and discussions. A short summary and conclusions complete the article.

## Many-Body PIP Approach

To begin, recall that the generic many-body representation of the potential, *V*, is given by
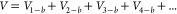
1where “*b*” represents a “body” and where “body” is yet to be defined and the definition of the body is a critical aspect of the representation. Also, each term on the right-hand side of eq 1 is a sum over all possible interactions of the body, starting with all 2-body interactions, *V*_2–*b*_, etc. *V*_1–*b*_ is a sum over isolated, noninteracting bodies.

For noncovalent interactions this representation was first introduced for rare gas atoms and has been recently used for an accurate potential for Ar gas up to 3-b terms.^54^ In more complex applications, “body” is typically a molecule, for example, for water potentials the body is the H_2_O monomer. For the interested reader, details of this approach for water up to 4-b interactions are given in a recent Perspective assessing CCSD(T)-based water potentials.^55^ For mixed noncovalent cases, all possible combinations of *n*-b terms are considered. For example, for a single hydrated proton in water
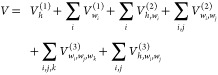
2In this expression, *V*_*h*_^(1)^ is the potential energy surface of the monomer H_3_O^+^, ,  and  are water 1-b, 2-b and 3-b interactions between water monomers. The 2-b interaction between H_3_O^+^ and H_2_O is denoted by  and  denotes the 3-b interaction between hydronium and water monomers. Details of this representation, where each term is at the CCSD(T) level, are given in ref (56). In the above example, each *n*-b interaction is represented by precise fits to thousands of CCSD(T) energies using PIPs. This representation is transferable since it can be applied to an arbitrary number of “bodies”, broadly defined. The accuracy clearly depends on the level of truncation of the expansion and this has to be checked on a case-by-case basis.

For general covalent interactions, the many-body representation is largely unexplored. It has been used to represent chemical reactions involving three and four atoms in pioneering work of Murrell et al.^57^ It has also been used implicitly in the fragmentation scheme of Collins et al.^58^ where the potential of a molecule is represented by a sum of energies of fragments. Thus, the nature and number of “*n*-body” terms depends on the details of the fragmentation scheme and so this is qualitatively different from the noncovalent many-body approach. (Different fragmentation schemes provide differing levels of accuracy compared to direct electronic energies of the full molecule.)

Paesani and co-workers reported a recent rediscovery and significant advancement of the fragmentation approach and tested it for small hydrocarbons.^9^ Specifically, the potential for linear butane is given by the sum of two CH_3_, two CH_2_ monomer energies plus two CH_3_–CH_2_ and one CH_2_–CH_2_ 2-b interaction energies, plus two adjacent CH_3_–CH_2_–CH_2_ 3-b interaction energies and one 4-b CH_3_–CH_2_–CH_2_–CH_3_ interaction energy. These terms were obtained from precise PIP fits to MP2 energies. Finally, a polarization term is added to account for nonbonded interactions. This approach is a major advance in two respects. First, the scheme is systematic and, as shown, the electronic energies of butane could be fit precisely. Second, the scheme is transferable to larger linear alkanes and tests up to decane were very promising.

We recently reported a different many-body expression for the linear alkane C_14_H_30_,^35^ up to and including 4-b interactions. Specifically, the expression for the potential is the same as eq 1, where now *V*_1–*b*_ is the total energy of all isolated carbon and hydrogen atoms. The 2-b interaction is given by
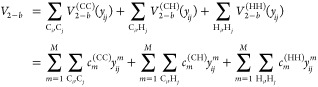
3where each 2-body term is just a linear expansion using a basis of powers of Morse variables for the corresponding CC, CH, and HH internuclear distances . We used the same total polynomial order *M* for all the 2-b terms, but they can be different for CC, CH, and HH. The 3-b term, *V*_3–*b*_ is given explicitly by the sum of 4 distinct 3-b interactions, namely
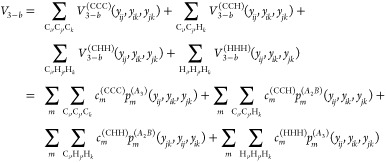
4where now the permutational symmetries of the two 3-b PIPs are *A*_3_ and *A*_2_*B*. Note that although CCC and HHH both use the same *A*_3_ PIPs, they use two different sets of coefficients; the same is true for CCH and CHH. Finally, the 4-b term, *V*_4–*b*_, is given by five distinct terms, namely
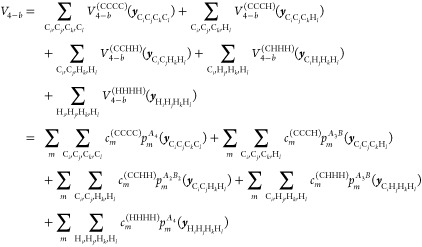
5where ***y***_*ijkl*_ is the collection of the 6 Morse variables in the tetramer formed by atoms *i*, *j*, *k*, and *l*.

To summarize, the general permutational symmetries of the PIPs bases are *A*_4_, *A*_3_*B*, *A*_2_*B*_2_, *A*_3_, and *A*_2_*B*. Also, it is important to note that the distinct 2-b, 3-b and 4-b PIP bases can have different range parameters for the corresponding Morse variables, and different maximum polynomial orders. Therefore, while the 2-b bases (i.e., powers of *y*_CC_, *y*_CH_, *y*_HH_) may appear to be included in 3-b and 4-b bases, they are in fact not because of different range parameters. Once these bases are set up, the linear coefficients are {***c***^(CC)^, ***c***^(CH)^, ***c***^(HH)^, ***c***^(CCC)^, ***c***^(CCH)^, ***c***^(CHH)^, ***c***^(HHH)^, ***c***^(CCCC)^, ***c***^(CCCH)^, ***c***^(CCHH)^, ***c***^(CHHH)^, ***c***^(HHHH)^} from eqs 3–5, and they are optimized all at once in an overdetermined linear least-squares fit to the total energies of this molecule.

This expression is general for all alkanes (and potentially for any molecule consisting of two elements). In the present targeted application, we train the potential on C_14_H_30_ using 253,646 B3LYP/cc-pVDZ energies, spanning a range from zero at the global minimum to roughly 230 kcal/mol. Details of this data set and fit have been published,^35^ with small modifications in this work. In that paper, the standard train/test protocol was used and the errors for both training and testing sets were very similar, indicating no overfitting.

The essential aspects of the fit are as follows. There are several hyperparameters in the model. First, we applied distance-based cutoffs for the many-body expansion, that is, if the largest distance in an *n*-mer exceeds the cutoff distance, the *n*-b energy from that *n*-mer is neglected when computing the total energy of the molecule. The polynomial orders used for *n*-b, and the Morse range parameters that control how fast the potential decays, are also hyperparameters. The values of these hyperparameters were chosen based on our past experience (range parameters), chemical intuition (range-parameters and cutoff distances), and the trade-off between accuracy and computational cost (polynomial orders and cutoff distances). It is generally expected that 4-b is shorter-ranged than 3-b than 2-b, so we applied smaller Morse range parameter and cutoff distance for 4-b, and larger values for 2-b. The values chosen below produce excellent precision in the testing. The 1-b energy is simply the sum of the energies of isolated carbon and hydrogen atoms computed using B3LYP/cc-pVDZ theory and it is not a trainable parameter. For the 2-b, a single Morse range parameter of 2.5 bohr was used, with a maximum power of 10, so the total number of 2-b coefficients is 30. The cutoff distance for 2-b is 18.0 bohr. For the 3-b, the Morse range parameter is 1.8 bohr, and the maximum polynomial order of the PIPs is 8. A switching function is applied when the maximum distance in a trimer is between 12.3 and 14.2 bohr, and the energy contribution is 0 when the maximum internuclear distance in the trimer is beyond 14.2 bohr. For the 4-b bases, the Morse range parameter is 1.2 bohr, and the maximum polynomial order is 6. The switching range for 4-body is between 8.5 and 10.4 bohr. All the bases are purified,^59,60^ that is, any polynomial that does not go to zero when an atom is infinitely far away from the remaining atoms is removed from the bases. Using the polynomial orders mentioned above, there are 32, 78, 78, 32, 40, 115, 174, 115, and 40 PIPs for CCC, CCH, CHH, HHH, CCCC, CCCH, CCHH, CHHH, HHHH, respectively. These sum up to 734 undetermined linear coefficients, including the 30 from all the 2-b interactions. All these linear coefficients were determined simultaneously in a single least-squares fit to ab initio energies of C_14_H_30_. The training root-mean-square error (RMSE) on energies is 0.33 kcal/mol (0.007 kcal/mol per atom). This is very small precision errors compared to typical ML values for such a large molecule and data set.

**Figure 1:**
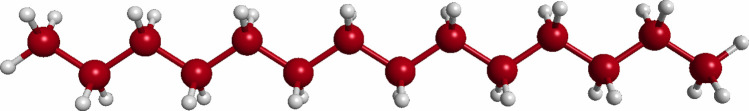
Global minimum structure of the alkane C_14_H_30_.

To test the transferability of this fit we considered a large range of linear alkanes, from butane to C_30_H_62_. The data sets for these are described in the next section, followed by the tests and results of the fidelity of the t-MB-PIP PES, trained on C_14_H_30_ (Figure 1).

## Computational Details

The testing set data comprises energies for molecules  where *n* = 4, 5, ..., 30. The global minimum energy of each molecule was found via geometry optimization at the B3LYP/cc-pVDZ level of theory. Next, a set of 200 initial configurations was obtained from configurations generated via molecular dynamics (MD) using the MM3(2000) force field^43,61^ and the Tinker 8 package:^62^ (10 ns simulation time, 2000 K, constant temperature Nosé-Hoover thermostat, 1 fs step size). Initial configurations for geometry optimization were selected by sampling MD configurations uniformly in energy over the energy range explored in the MD simulations. Each of these 200 initial configurations was then optimized at the B3LYP/cc-pVDZ level of theory and vibrational frequencies were calculated to characterize the stationary points. Geometries and associated energies from each step in the optimization procedure were collected to form the testing set. A total of 178,842 configurations were generated using this procedure. As larger molecules generally require more optimization steps, the number of testing geometries/energies generated via this procedure varies from ≈2800 for C_4_H_10_ to ≈10,100 for C_30_H_62_. Next, we removed geometrically similar configurations when their energy difference is smaller than 1 cm^–1^. As for the energy range for each alkane, we took note of the fact that training was done for C_14_H_30_ up to 80,000 cm^–1^ and so we scaled that range appropriately for each alkane. Thus, the energy maximum for each alkane is given by (80,000/14) × *N*_*c*_, where *N*_*c*_ is the number of carbon atoms. And we multiplied that number by 1.25 to extend the range by 25%. The final testing data used for the correlation plots in the next section consist of 1290 geometries/energies for C_4_H_10_, 2997 for C_13_H_28_, 3842 for C_21_H_44_, and 4899 for C_30_H_62_. All density functional theory (DFT) calculations were performed using the Gaussian 16∇ computational chemistry package.^63^

For each molecule, geometry optimization and normal-mode analysis using the t-MB-PIP PES were carried out at the linear all-*trans* minimum. The harmonic frequencies from the t-MB-PIP PES are compared with B3LYP/cc-pVDZ frequencies.

Two microcanonical (NVE) molecular dynamics trajectories were calculated for C_30_H_62_ using the PES. The molecular dynamics simulation employed a time step of 5 au, or about 0.121 fs per step, for 30,000 steps. In both applications, trajectories were initiated at the linear all-*trans* minimum of the potential, and a total energy of 5000 and 80,000 cm^–1^ (14.3 and 229.2 kcal/mol) was specified and distributed randomly among the kinetic energies of all atoms. We chose a low total energy of 5000 cm^–1^, which produces a sharp spectrum at the linear minimum, and a higher energy of 80,000 cm^–1^ where anharmonic large amplitude effects are seen. The total angular momentum was set to be zero for each trajectory. We use the standard velocity-Verlet algorithm and energy is well conserved during all MD simulations to ca. 1%. Power spectra were computed using the last 20,000 steps of the two trajectories on an open-source web platform “SEMISOFT”.^64−66^

## Results

**Figure 2:**
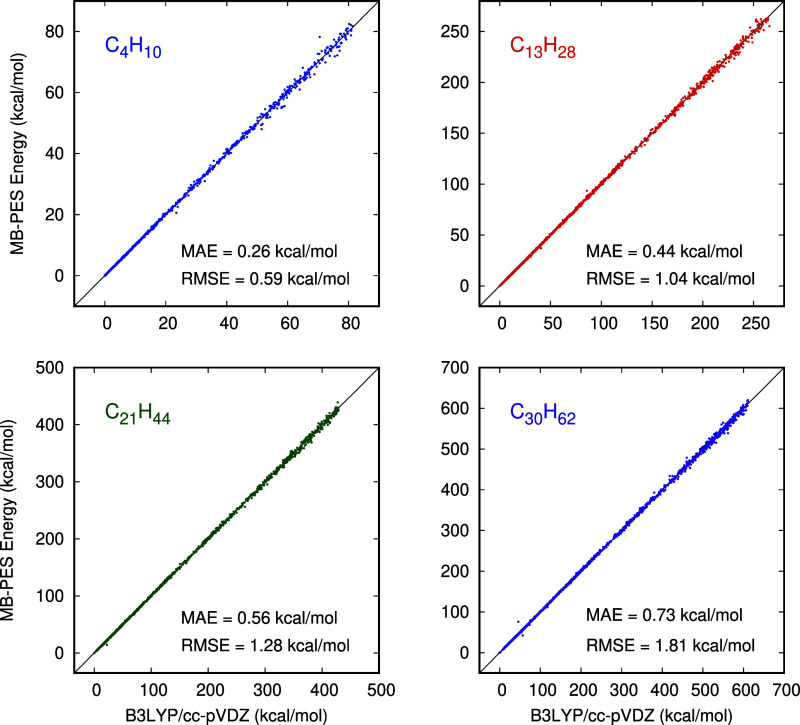
Correlation plots of the energies for the indicated alkane from the t-MB-PIP PES and direct B3LYP calculations.

To begin, we present standard correlation plots for energies in Figure 2 for four selected linear alkanes, C_4_H_10_, C_13_H_28_, C_21_H_44_, and C_30_H_62_, which span the full range of linear alkanes considered from the t-MB-PIP PES and direct B3LYP calculations. In these plots note that, as expected, the energy range scales roughly linearly with the size of the alkane. However, the MAE and RMSE scale sublinearly. On a per/atom basis the RMSEs are 0.042, 0.025, 0.020, 0.020 kcal/mol for the four alkanes shown. These are both nearly constant and remarkably small, as discussed in detail in the next section.

**Figure 3:**
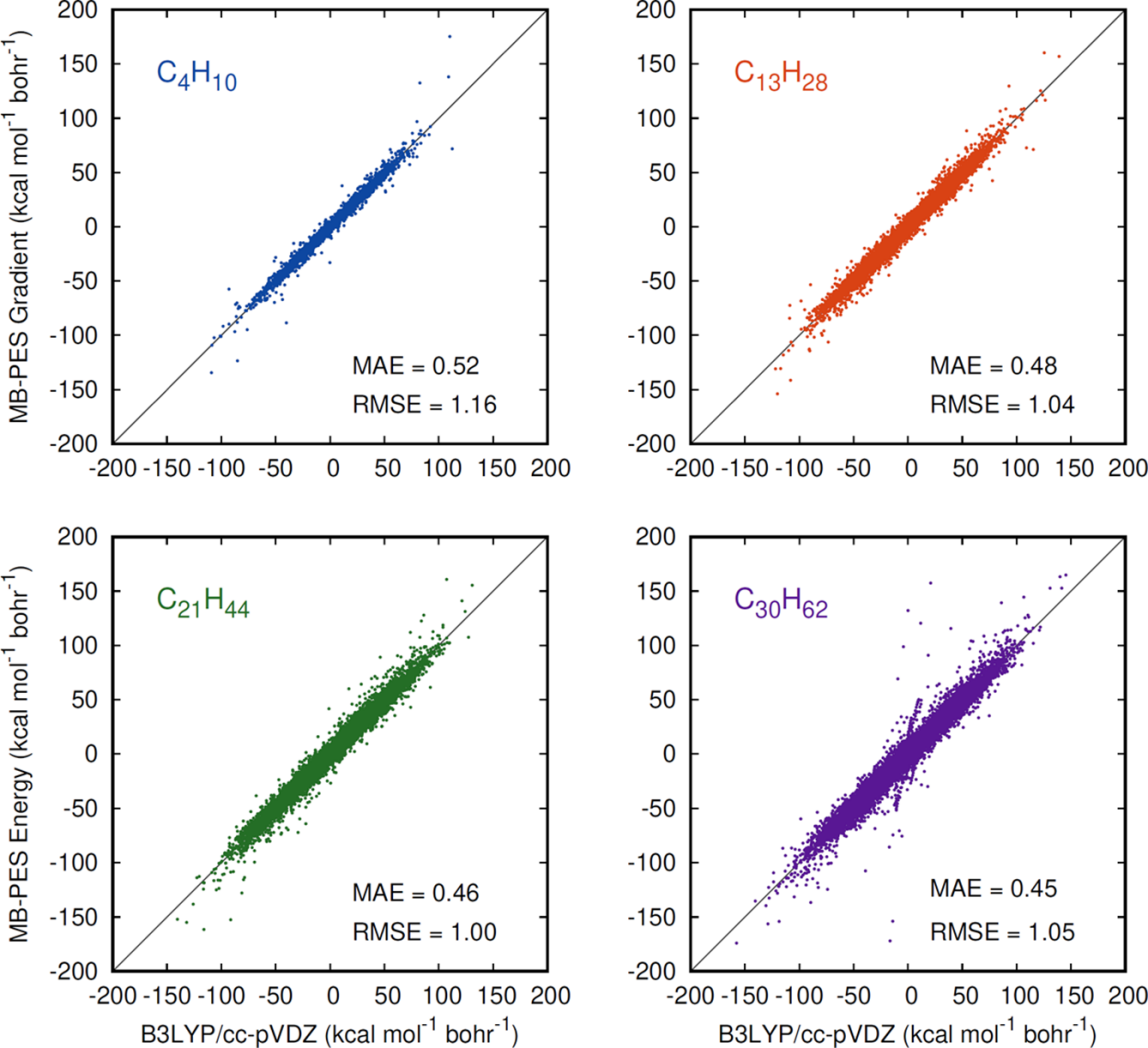
Correlation plots of the gradients for the indicated alkane from the t-MB-PIP PES and direct B3LYP calculations.

Next, we show corresponding correlation plots for gradients in Figure 3, and recall that the training data set does not include gradients. As seen, the MAE is roughly 0.5 kcal mol^–1^ bohr^–1^ and shows a slight decrease with increasing alkane size. However, as seen, there is significantly more scatter in these plots, than in the energy ones, especially for C_30_H_62_ and notably where the DFT gradient is zero. These are high-energy stationary points, minima or saddle points and the imprecision in getting these for the alkane is almost certainly due to the absence of training on gradients.

**Figure 4:**
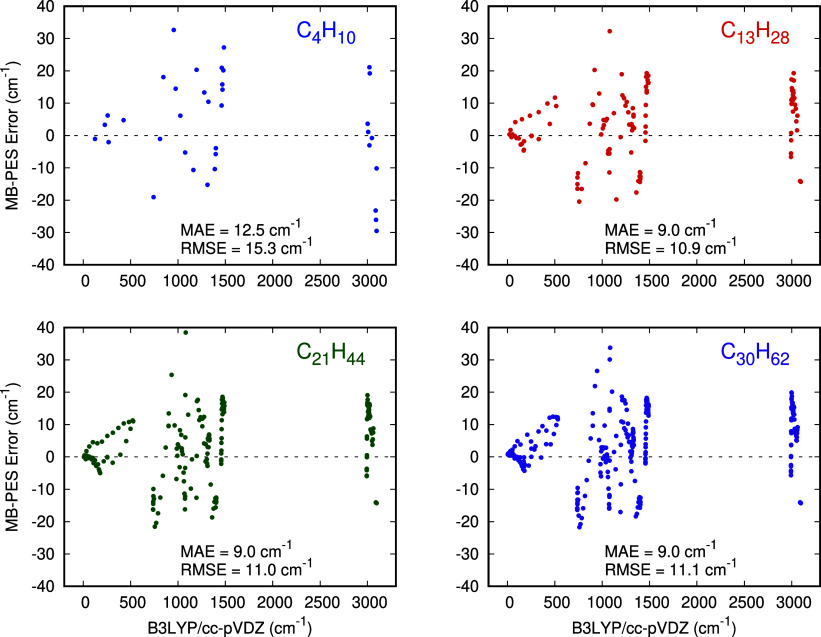
Errors in the harmonic frequencies (in cm^–1^) from the t-MB-PIP PES compared with direct B3LYP/cc-pVDZ harmonic frequencies. Each point in the plots represents a normal mode; *x*-axis is the harmonic frequency at the B3LYP/cc-pVDZ level of theory, and *y*-axis measures the difference between the PES frequency and the ab initio one. Both are obtained from diagonalization of the corresponding mass-weighted Hessian at the linear all-*trans* minimum.

Figure 4 shows the errors and overall MAE and RMSE of the t-MB-PIP harmonic frequencies relative to the B3LYP/cc-pVDZ ones for the indicated alkanes at their respective linear all-*trans* minimum. These are in range of 10 cm^–1^ and virtually unchanged from butane to C_30_H_62_. These metrics are very similar to the error of the trained alkane (C_14_H_30_) of 13.7 cm^–1^. This is further evidence of the excellent performance of the transferability of the MB-PIP PES.

**Figure 5:**
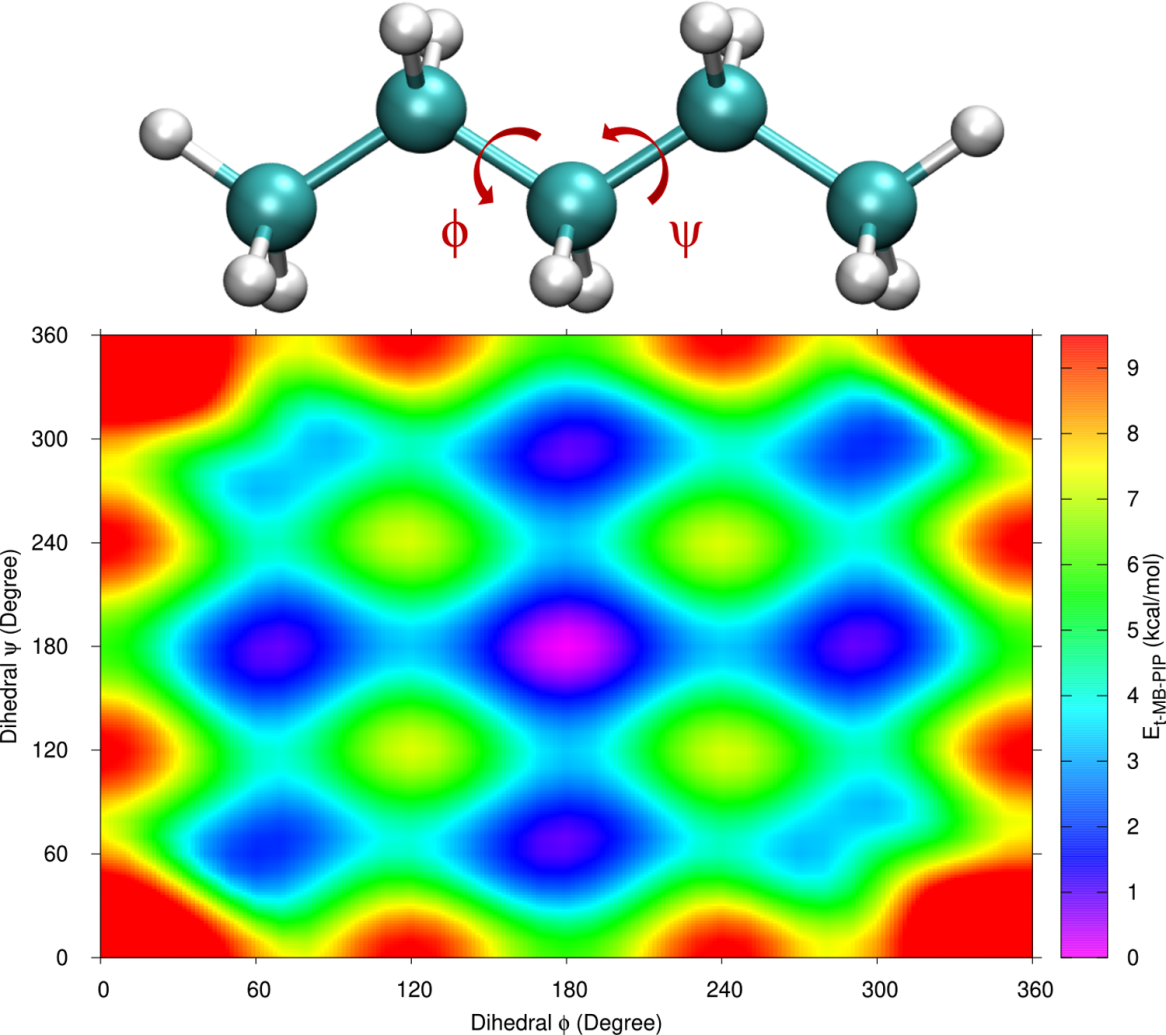
Relative energy (kcal/mol) of *n*-pentane from the t-MB-PIP PES as a function of two backbone dihedral angles. Values above 9.5 kcal/mol have been clipped for better contrast.

To further test the accuracy of this transferable PES for linear alkanes, we compare the torsional barriers of pentane from the PES with CCSD(T) benchmark values calculated by Martin.^67^ Specifically, we located 14 stationary points by varying two dihedral angles: C1–C2–C3–C4 and C2–C3–C4–C5. Following the conventional notation for the dihedral angles, i.e., *t* = trans ≈ 180°, *g* = gauche ≈ ± 60°, *x* = cross ≈ ± 90°, γ ≈ ± 75°, ξ = skew ≈ ± 120°, τ = eclipsed ≈ 0°, the energies and the dihedral angles of the 14 stationary points from the t-MB-PIP PES are summarized in Table 1, alongside CCSD(T) benchmarks and B3LYP/cc-pVTZ results. The energies and dihedral angles of the t-MB-PIP PES are in good agreement with those of B3LYP, as expected, since the PES was trained on B3LYP/cc-pVDZ energies of C_14_H_30_. Furthermore, the t-MB-PIP PES and B3LYP results are also in good agreement with the benchmark CCSD(T) results. Last, we show the contour plot of the pentane torsional surface as a function of the two dihedral angles ϕ and ψ in Figure 5. For this contour plot, the geometries of the grid points were taken from ref (67), which were obtained using constrained optimization at SCS-MP2/cc-pVTZ level of theory. The t-MB-PIP PES is directly applied to calculate the energies on these grid points.

**Table 1:** Relative Energies (kcal/mol) of the 14 Stationary Points on the *n*-Pentane Torsional Surface from t-MB-PIP PES and Various Levels of Theory

	energy (kcal/mol)			dihedral angles (degree)		
structure^a^	CCSD(T)^b^	PES	B3LYP	CCSD(T)^b^	PES	B3LYP
*tt* MIN	0.00	0.00	0.00	(180.0, 180.0)	(180.0, 180.0)	(180.0, 180.0)
*tg* MIN	0.58	0.85	0.91	(176.2, 64.3)	(178.6, 66.9)	(177.3, 66.2)
*gg* MIN	0.91	1.76	1.66	(58.7, 58.7)	(60.2, 60.2)	(63.7, 63.7)
*gx*^–^ MIN	2.76	3.10	3.45	(60.5, −94.1)	(62.4, −86.8)	(65.2, −90.4)
*t*ξ^–^ TS	3.11	3.24	3.08	(179.0, −119.7)	(178.5, −119.7)	(179.3, −118.8)
γγ^–^ TS	3.17	3.27	3.56	(76.1, −76.1)	(76.2, −76.2)	(77.2, −77.2)
*g*ξ^–^ TS	3.35	4.04	3.85	(67.1, −122.0)	(65.4, −121.2)	(66.7, −118.7)
*g*ξ TS	3.48	3.96	3.82	(69.0, 117.7)	(67.8, 117.1)	(69.4, 117.7)
*t*τ TS	5.38	5.76	5.72	(180.0, 0.0)	(180.0, 0.0)	(180.0, 0.0)
τ*x* TS	7.05	7.31	7.57	(−5.3, 82.4)	(−4.6, 78.8)	(−4.9, 81.4)
ξξ^–^ SP	7.14	7.10	6.98	(119.0, −119.0)	(119.8, −119.8)	(118.3, −118.3)
ξξ SP	7.18	7.20	6.90	(120.6, 120.6)	(120.7, 120.7)	(120.2, 120.2)
ξτ SP	9.32	9.65	9.44	(120.9, 1.3)	(120.5, 0.1)	(120.2, 0.8)
ττ SP	16.66	16.78	16.78	(0.0, 0.0)	(0.0, 0.0)	(0.0, 0.0)

a MIN = minimum, TS = first-order saddle point, SP = second-order saddle point.

b From ref (67).

**Figure 6:**
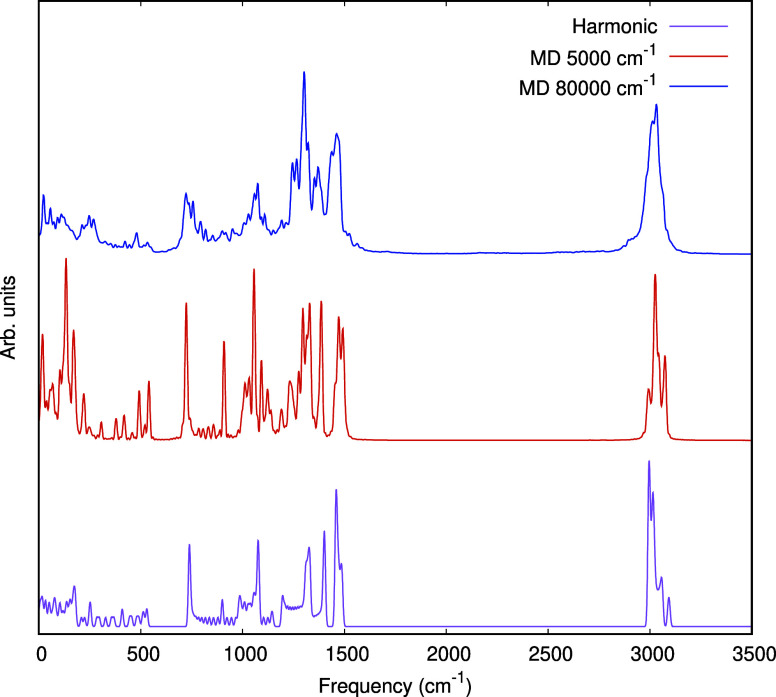
Power spectra of C_30_H_62_ using MD trajectories run at a low total energy of 5000 cm^–1^, which produces a sharp spectrum at the linear minimum, and the higher energy of 80,000 cm^–1^, where anharmonic large amplitude effects are seen. The harmonic power spectrum at the B3LYP/cc-pVDZ level of theory is also shown.

Next as a test at the other extreme, i.e., C_30_H_62_, we show the vibrational power spectra computed from two microcanonical trajectories, at the indicated total energies, in Figure 6, as well as the harmonic power spectrum at B3LYP/cc-pVDZ level of theory. The main objective in showing these is to indicate the robustness of the t-MB-PIP PES for this large alkane. However, we do note that the spectrum computed with 5000 cm^–1^ MD is in reasonably good agreement with the power spectrum based on B3LYP/cc-pVDZ harmonic frequencies, obtained as follows. First, each normal mode was represented as a stick with unity intensity. Then each stick was broadened using a Gaussian function with σ = 5.0 cm^–1^ (corresponding to a full width at half-maximum of 11.8 cm^–1^). The dominant band at around 3100 cm^–1^. This is basically the collection of CH-stretches, and the triplet structure seen for the 5000 cm^–1^ trajectory is very similar to one seen experimentally at room temperature for C_15_H_32_ and calculated by us for C_14_H_30_.^36^ The bands in the region of 1200–1500 cm^–1^ correspond to bends, and the lowest frequency bands correspond to “acoustic” modes of the CC backbone. More detailed analysis of these bands would certainly be of interest.

**Figure 7:**
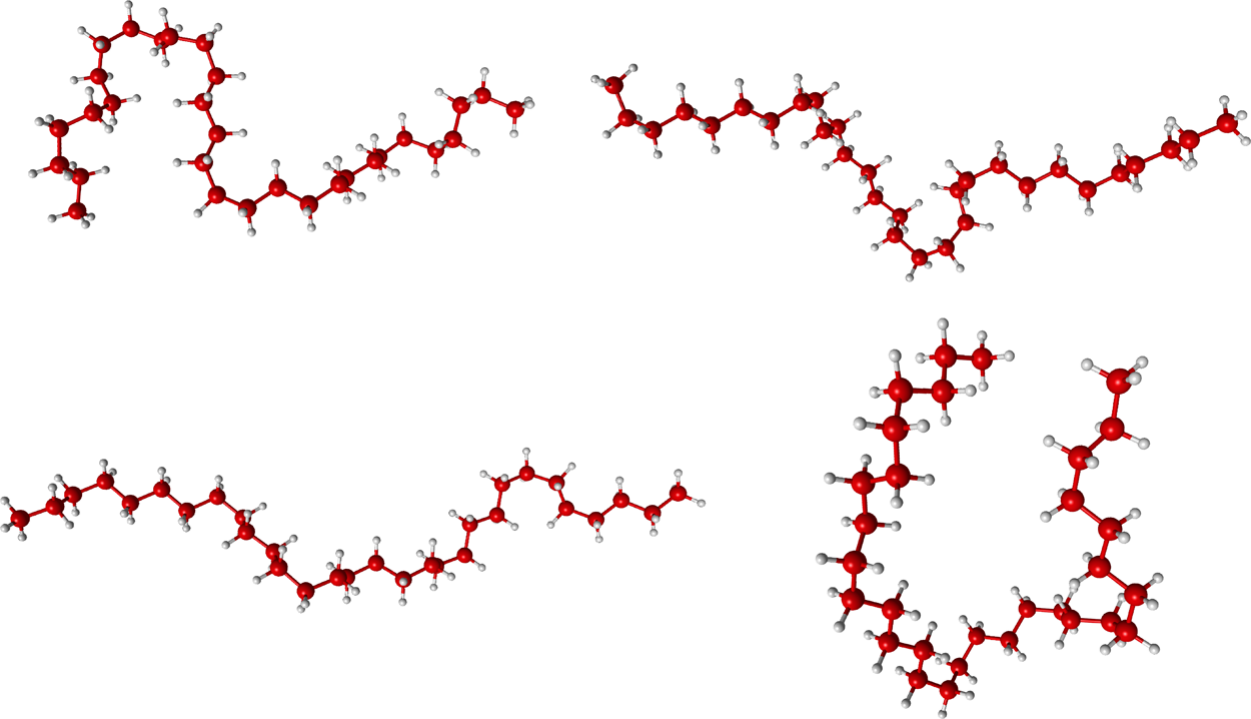
Sample of four C_30_H_62_ configurations.

Finally, to indicate the extent of alkane flexibility, four configurations of C_30_H_62_ from the correlation plot for this alkane are given in Figure 7. As seen, these are highly diverse, and just a reminder of the high flexibility of this and all large alkanes.^35,36^

## Discussion

As noted in the Introduction, many-body atom-centered approaches to energies are in principle transferable, with the earliest work for atomic solids, notably Si, dating back to the mid 1980s.^68,69^ More recent, machine-learned atom-centered methods now abound in the literature, with perhaps the ANI approach, an elaborated version of the Behler-Parinello atom-centered Neural Network method, being among the first and most prominent across a large class of molecules.^21,45,70^ Of relevance to the present work are the aPIPs, ACE and most recently MACE.^17,18,47,71,72^ These all borrow from the atomic cluster expansion,^47^ which is based on an atom-centered expansion, where atoms interact with neighbors via 2-b, 3-b, and higher-order potentials. The basis for these *n*-b terms are given in terms of internuclear distances and angles and overall are more complex than the PIPs bases we use here, which are just functions of the transformed internuclear distances (Morse variables). A somewhat related approach that uses a basis of B-splines was proposed for an atom-centered expansion up to 3-b terms and applied to tungsten.^71^ This basis is fast to evaluate; however, it is not permutationally invariant.

The method that is perhaps closest to the one developed here, and for which data on fitting small alkanes is available, is the aPIPs approach.^17,18^ (This is a successor to our PIPs approach.) The method was successful in terms of precision and speed relative to other machine-learned many-body approaches for materials and molecules. However, it was not pursued further by those authors. Finally, we note that while the ACE and present MB-PIPs methods are polynomial linear regression methods, the former belongs to the class of underdetermined least-squares, i.e., more linear coefficients than data, whereas MB-PIPs (and all of our PIPs fits) are overdetermined, i.e., fewer linear coefficients than data. Another important difference is a formal one, namely aPIPs and ACE represent the total energy as the sum of atomic energies instead of the many-body expansion given above.

All of these approaches aim for universality, in contrast to the targeted approach we take here. As we showed above, the targeted approach for alkanes achieves excellent results, as determined by the high and nearly constant precision over a large range of alkanes. Our expectation is that the targeted approach should achieve higher performance than universal ones.

**Figure 8:**
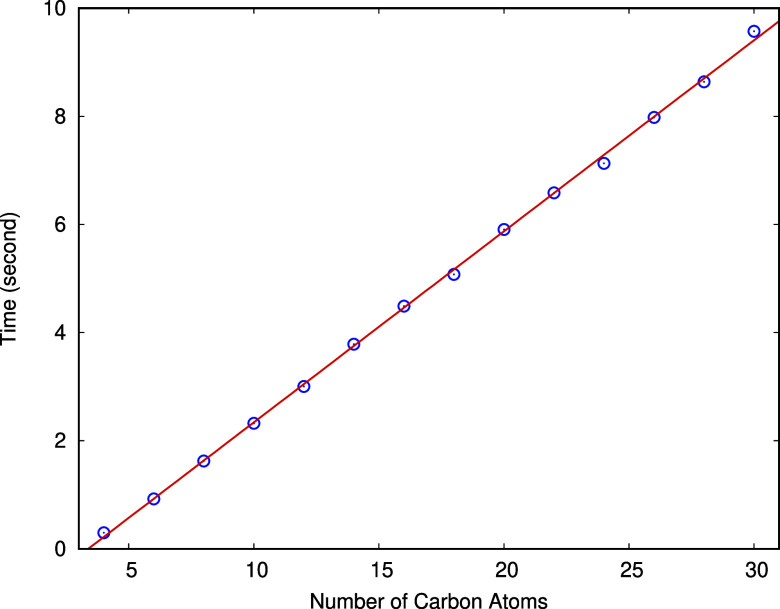
Computational time to evaluate the energies and gradients of 1000 geometries using t-MB-PIP PES for alkanes with different number of carbon atoms. The open circles are the actual timings, and the solid line is a linear fit.

Next we consider the timings to predict the energies and gradients for alkanes with different number of carbon atoms using the PES. The number of *k*-b terms scales as , where *n* is the number of atoms, if no cutoff is applied. With a distance-based cutoff, however, the MB-PIP approach effectively achieves linear scaling, as is shown in Figure 8. The open circles represents the computational time to evaluate the energies and gradients of 1000 geometries using a single core on an Intel i7-12700K CPU, and solid line is a linear fit to the data. Therefore, this approach is applicable to much larger systems due to this friendly linear scaling.

We conclude this section with comments about future work in progress. One is to investigate adding dispersion to the present B3LYP energies. Several such corrections, such as D3,^73^ D4,^74^ and the many-body dispersion^75^ being explored. In another one, we are investigating the Δ-ML approach that we have used for a number of PIP PESs,^55,76,77^ to correct the current DFT-based t-MB-PIP potentials. The idea is to note that Δ-ML can be applied to each *n*-b term in eq 1. This can be achieved up to 4-b terms with an alkane as small as butane, for which CCSD(T) calculations are very feasible. This approach is currently underway and we hope to report results soon. Training on a broader class of alkanes, including branched and cyclic ones is certainly now possible, given the appropriate data sets.

In the other work in progress, Yu et al. have investigated a novel MB-PIP-NN approach, thus far on noncovalent interactions, and have used it to achieve excellent performance using just 1 and 2-b terms. In this case the body is a molecule and the 1-b and 2-b terms are inputs to standard neural networks.^78^ This approach is inspired by the success of PIP-NN for molecules and molecule-rigid surface applications,^37,79−81^ and the related FI-NN approach.^39,82,83^

## Summary and Conclusions

A transferable, targeted many-body permutationally invariant polynomial force-field for linear alkanes has been tested for a range of alkanes from butane to C_30_H_62_. The training for this transferable potential was done in a recent paper focused on C_14_H_30_. The transferability is of unprecedented precision and the same as the high precision of the trained MB-PIP PES. Excellent agreement was reported with previous B3LYP and close agreement with benchmark CCSD(T) “ϕ-ψ” energies of pentane. These all demonstrate that the targeted transferability is an excellent compromise between “molecule-specific” machine learning and “universal” transferability. The present approach is clearly applicable to other targeted examples, for example to a subset of amino acids containing H, C, O, and N, and to alcohols, etc.

Finally, we note that all the data sets, including the one for C_14_H_30_ are available (see next section) for those who may wish to test other machine learned methods for potentials.

## Data Availability

The present t-MB-PIP PES for linear alkanes is publicly available at https://github.com/szquchen/alkane_PES. The geometries, energies, and gradients for all of the test geometries used in this article are available at https://github.com/jmbowma/QM-22/tree/main/C14H30/testing. The data set for the C_14_H_30_ alkane is available at https://github.com/jmbowma/QM-22.

## Footnotes

# Commonly reported energy units are used in this paper. These may be converted to SI units using the equivalences 4.184 kJ/mol = 1 kcal/mol = 350 cm^–1^ = 43 meV.

∇ Certain commercial software is identified in this paper to foster understanding. Such identification does not imply recommendation or endorsement by the National Institute of Standards and Technology, nor does it imply that the software identified is necessarily the best available for the purpose.
